# Supplementary material for: Regulation of the evolutionarily conserved muscle myofibrillar matrix by cell type dependent and independent mechanisms
Source: Nat Commun. 2022 May 13;13:2661. doi: 10.1038/s41467-022-30401-9 (PMC9106682; doi:10.1038/s41467-022-30401-9)
Supplement: Supplementary file 1 — Supplementary Information [file 41467_2022_30401_MOESM1_ESM.pdf]

SUPPLEMENTARY INFORMATION

Regulation of the Evolutionarily Conserved Muscle Myofibrillar Matrix by Cell Type Dependent and Independent Mechanisms

Relative Expression of Myofibrillar Network Regulators and Sarcomere Branching Frequency in Drosophila Muscles

	Leg	TDT	IF	NCDN KD IF	Salm KD IF
**Salm**	-	+	++	++	-
**H15**	+	+++	++	++	-
**NCDN**	+++	++	+	-	++++
**Sarcomere Branching**	++	+	-	++	++

Supplementary Table 1: Salm expression based on immunofluorescence analyses from[1-3] and Supplementary Figure 5 here. H15 expression based on mass spectrometry analyses from[2] and Supplementary Figure 5 here. NCDN expression based on mass spectrometry analyses from[2] and Supplementary Figure 4 here. Sarcomere branching frequencies from Figures 1, 3, and 4 here.

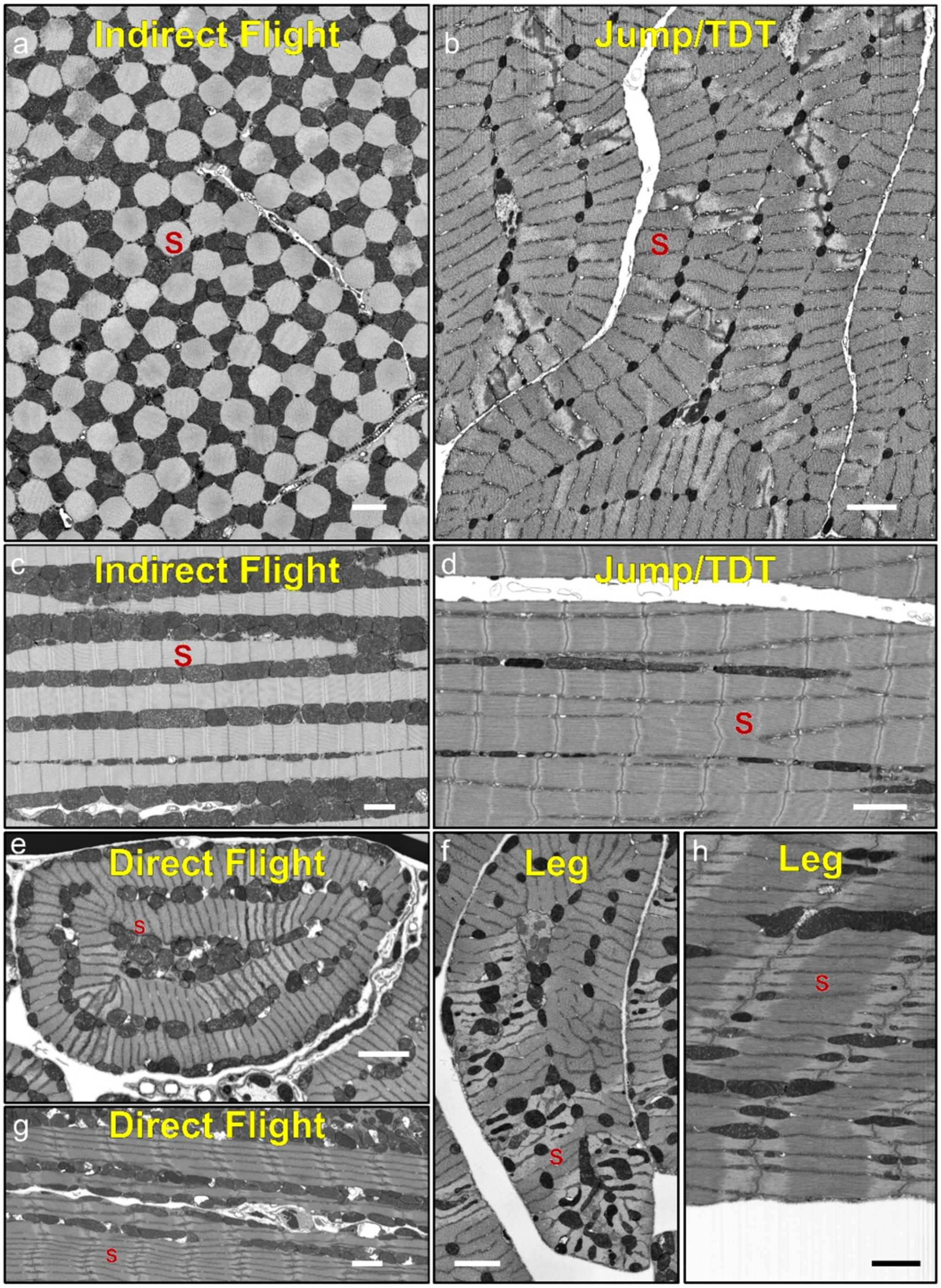

Supplementary Figure 1: The variety in size, shape, and content of the contractile apparatus and surrounding organelles across *Drosophila* muscles. Representative scanning electron microscopy images of Drosophila muscle longitudinal and cross-sections. Red S in each image signifies a single sarcomere. Images are representative of more than one hundred per muscle type. Scale bar – 2 µm.

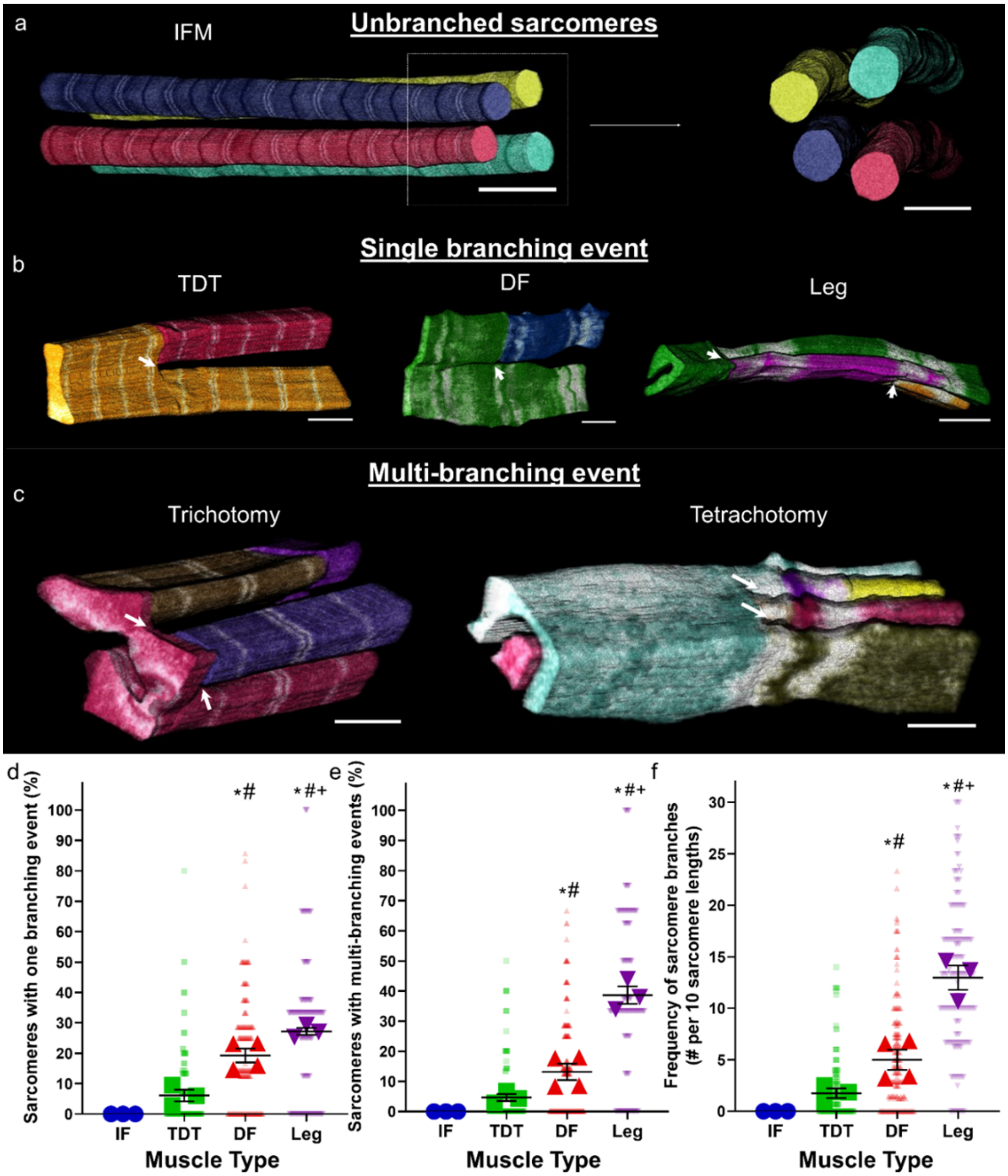

Supplementary Figure 2: Types of events within a sarcomere. a) 3D rendering of longitudinal and cross sections of isolated myofibrils in fibrillar indirect flight muscles. b) 3D rendering of single branching event in tubular muscles of TDT, DF, and leg muscles. c) 3D renderings of multi-branching events (more than one event) in tubular muscles. Examples of one sarcomere branching into three segments (Trichotomy) and into four segments (Tetrachotomy) are shown. For a-c, individual colors represent different myofibrillar segments. d) Percentage of sarcomeres with just one branching event in wild type muscles. e) percentage of sarcomeres with just one branching event in wild type muscles. f) Frequency of sarcomere branching in wild type muscles. N values for d, e, f: IF—3 muscle cells, 3 datasets, 114 myofibrils, 982 sarcomeres; TDT—3 muscle cells, 2 datasets, 160 myofibrils, 1863 sarcomeres; DF—4 muscle cells, 1 dataset, 200 myofibrils, 1461 sarcomeres; and leg—3 muscle cells, 2 datasets, 150 myofibrils, 690 sarcomeres. Larger shape symbols represent data from a single cell and smaller shape symbols represent data from a single myofibril. Bars represent muscle cell overall mean ± SE. Asterisk (*): Significantly different from fibrillar indirect flight (one-way ANOVA, $P < 0.05$). Pound sign (#): Significantly different from tubular TDT. Plus sign (+): Significantly different from tubular DF. Scale bars – 2 µm.

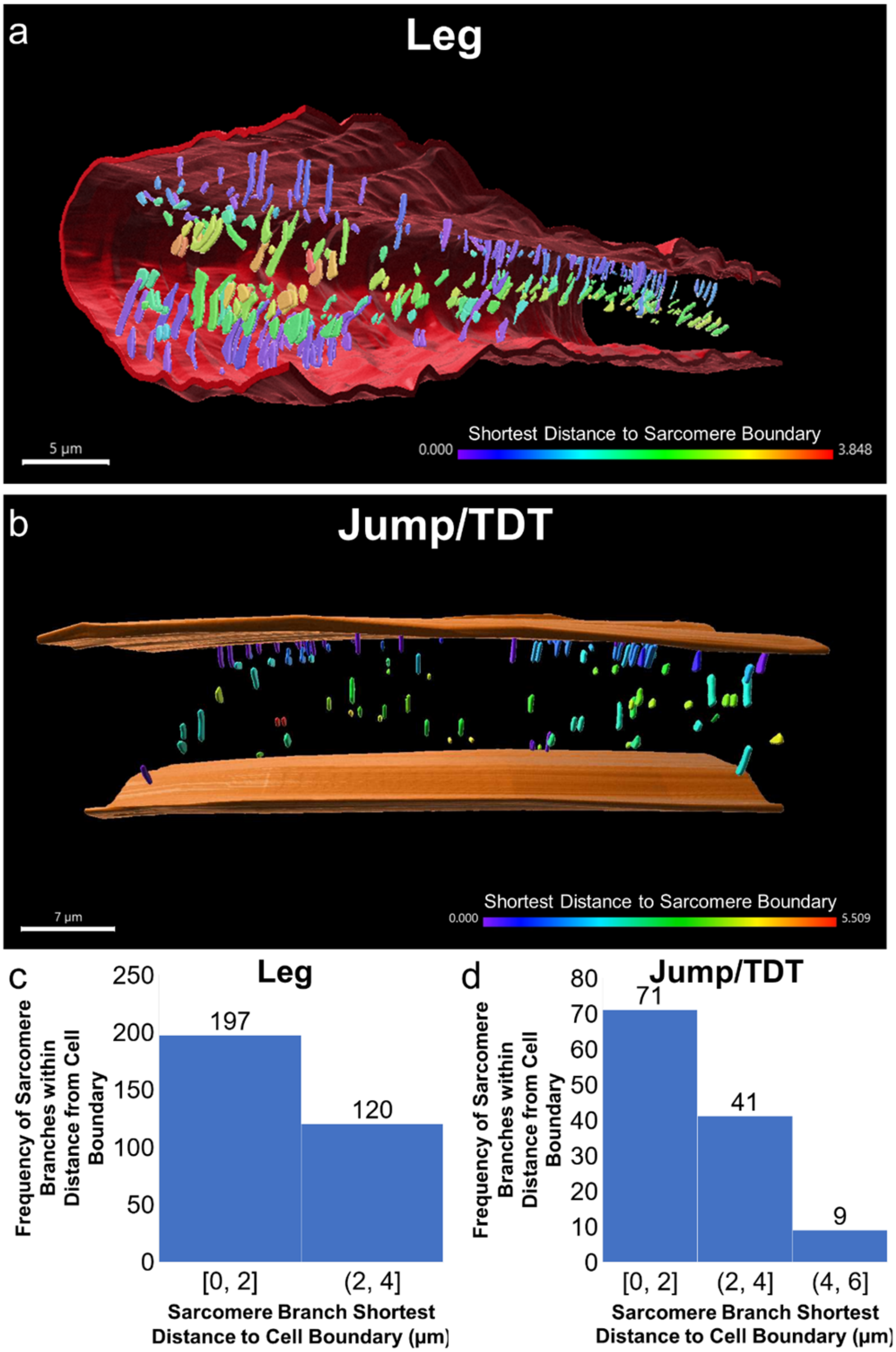

Supplementary Figure 3: Sarcomere branching occurs more frequently near the muscle cell boundary. a) 3D rendering of the leg muscle sarcolemma (red) and the location of the branch points within the muscle (colored based on distance to sarcolemma). b) 3D rendering of the jump muscle sarcolemma (orange) and the location of the branch points within the muscle (colored based on distance to sarcolemma). c) Histogram of the location of sarcomere branches in the leg muscle relative to the sarcolemma using 2 µm bins. d) Histogram of the location of sarcomere branches in the jump muscle relative to the sarcolemma using 2 µm bins.

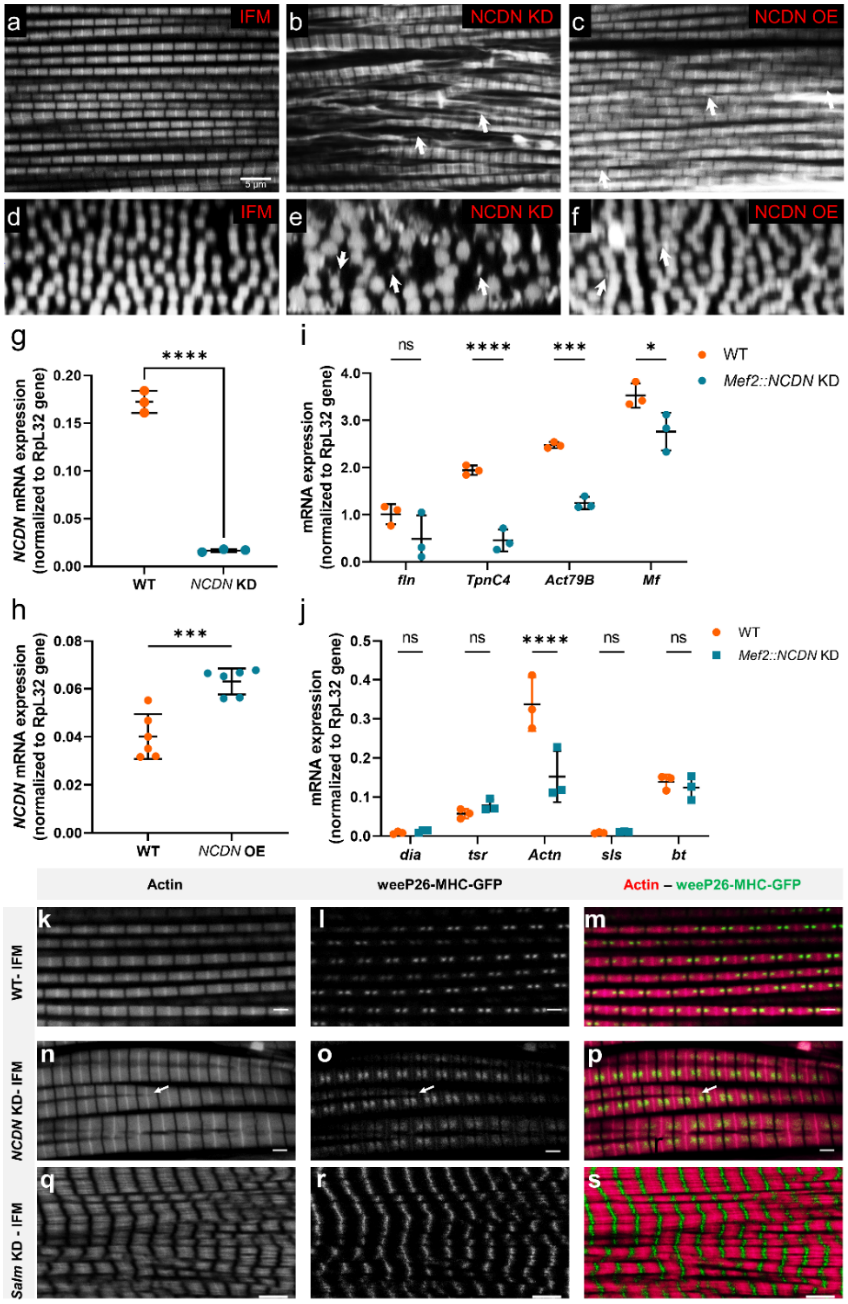

Supplementary Figure 4: Neurochondrin misexpression causes sarcomere branching. a-f Confocal microscopy images of wild type (WT, a,d) and *Mef2-Gal4* driven neurochondrin (*NCDN*) knockdown (KD, b,e) and overexpression (OE, c,f) adult indirect flight muscles (IFMs). Longitudinal (a-c) and cross-section (d-f) images are shown. White arrows highlight thin, branching myofibrillar segments. Representative of muscles from three flies. **g** NCDN mRNA expression relative to RpL32 in WT and *NCDN* KD IFMs. **h** NCDN mRNA expression relative to RpL32 in WT and *NCDN* OE IFMs. **i,j** Relative mRNA expression of contractile transcripts in WT and *NCDN* KD IFMs. **k-s** Confocal microscopy images of WT (k-m), *Mef2-Gal4*::*NCDN* KD (n-p), and *Mef2-Gal4::Salm* KD (q-s) IFMs stained with phalloidin (k,n,q) and tagged with weep26-MHC-GFP (l,o,r). Merged images are shown in m,p,s. White arrows highlight a sarcomere branch. Representative of muscles from three flies. **** p<0.001, *** p<0.001, * p<0.05 for two-sided, independent t-test (g,h) and one way ANOVA with Tukey's HSD post hoc test (i,j). Scale bars: 5µm. Error bars show mean ± SE for three samples (individual points) of 20 pooled thoraces per group.

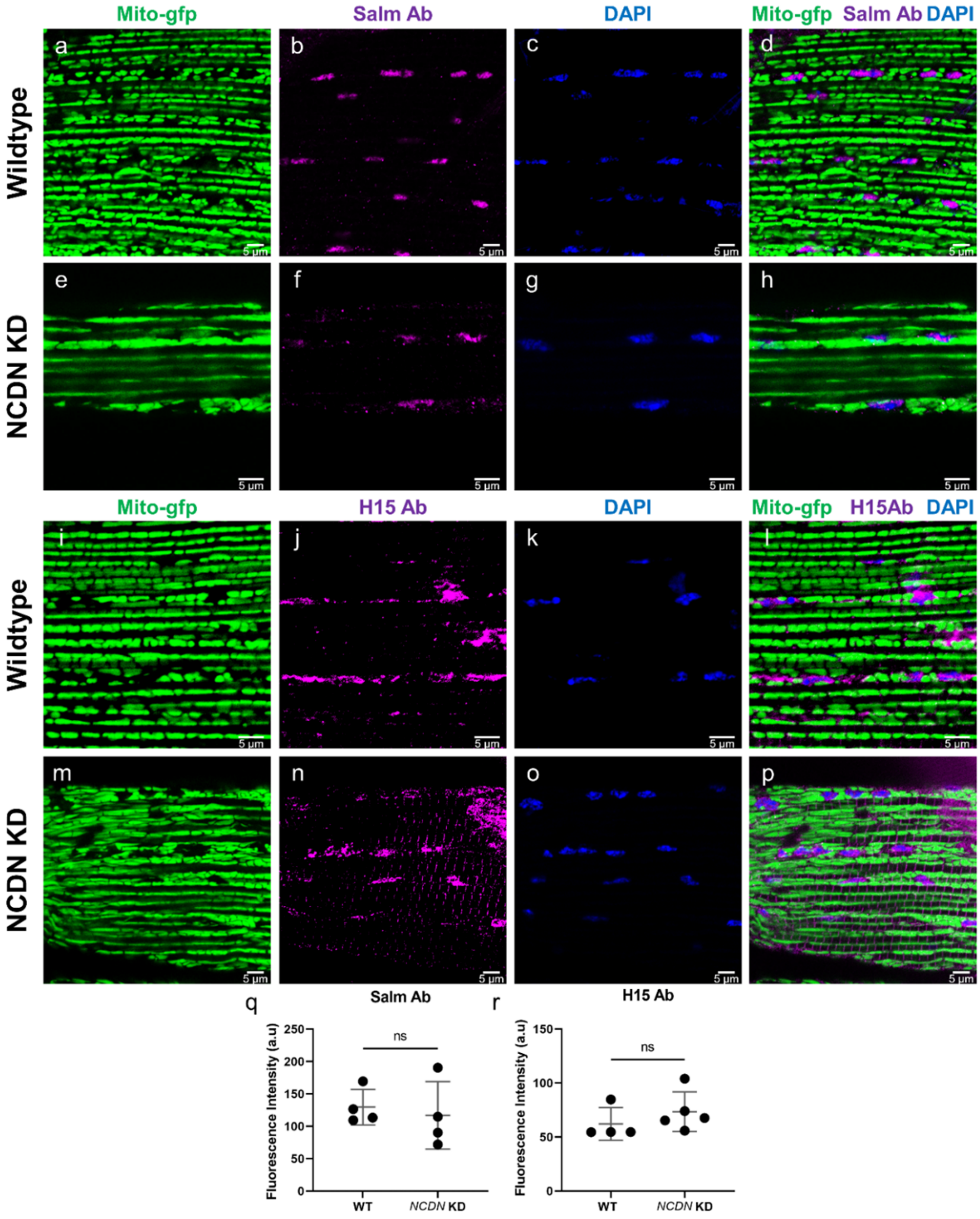

Supplementary Figure 5: ***Neurochondrin*** **knockdown does not alter indirect flight muscle fibrillar fate.** a-h) Locations of mitochondria (a,e), Salm protein expression (b,f) and nuclei (c,g) in wild type (a-d) and *neurochondrin* (*NCDN*) knockdown (KD) (e-h) indirect flight muscles as imaged under a confocal microscope. Representative of four muscle cells. i-p) Locations of mitochondria (i,m), H15 protein expression (j,n) and nuclei (k,o) in wild type (i-l) and *neurochondrin* (*NCDN*) knockdown (KD) (m-p) indirect flight muscles as imaged under a confocal microscope. Representative of four muscle cells. q) Quantification of Salm immunofluorescence in four cells each for wild type (WT) and *NCDN* KD muscles. (r) Quantification of H15 immunofluorescence in four WT and five *NCDN* KD cells. Circles represent individual muscle cells, bars equal mean ± SE, ns: P>0.05 in two-sided, independent t-test.

Supplementary References

1 Bryantsev, A. L. *et al.* Extradenticle and homothorax control adult muscle fiber identity in Drosophila. *Developmental cell* **23**, 664-673, (2012).

2 Katti, P., Ajayi, P. T., Aponte, A. M., Bleck, C. K. & Glancy, B. An Evolutionarily Conserved Regulatory Pathway of Muscle Mitochondrial Network Organization. *bioRxiv* **doi.org/10.1101/2021.09.30.462204**, (2021).

3 Schönbauer, C. *et al.* Spalt mediates an evolutionarily conserved switch to fibrillar muscle fate in insects. *Nature* **479**, 406-409, (2011).
