# Supplementary material for: Regulation of the evolutionarily conserved muscle myofibrillar matrix by cell type dependent and independent mechanisms
Source: Nat Commun. 2022 May 13;13:2661. doi: 10.1038/s41467-022-30401-9 (PMC9106682; doi:10.1038/s41467-022-30401-9)
Supplement: Supplementary file 3 — Description of Additional Supplementary Files [file 41467_2022_30401_MOESM3_ESM.pdf]

Description of Additional Supplementary Files

File Name: Supplementary Movie 1
Description: 3D rendering and fly through of the leg muscle myofibrillar network

File Name: Supplementary Movie 2
Description: 3D rendering and fly through of the jump muscle myofibrillar network

File Name: Supplementary Movie 3
Description: Representative raw data and 3D rendering example of a single branching event

File Name: Supplementary Movie 4
Description: Representative 3D rendering example of a multi-branching event

File Name: Supplementary Movie 5
Description: 3D rendering of the H15 knockdown TDT muscle Z-disks

File Name: Supplementary Movie 6
Description: Raw FIB-SEM data depicting a Z-disk hole in H15 knockdown TDT muscle

File Name: Supplementary Movie 7
Description: 3D rendering and fly through of the H15 knockdown TDT muscle myofibrillar network

File Name: Supplementary Movie 8
Description: 3D rendering and fly through of the salm knockdown indirect flight muscle myofibrillar network

File Name: Supplementary Movie 9
Description: 3D rendering of the salm knockdown indirect flight muscle Z-disks

File Name: Supplementary Movie 10
Description: Raw FIB-SEM dataset from neurochondrin knockdown flight muscles

File Name: Supplementary Movie 11
Description: 3D rendering and fly through of the neurochondrin knockdown indirect flight muscle myofibrillar network

File Name: Supplementary Movie 12
Description: 3D rendering of the neurochondrin knockdown indirect flight muscle Z-disks

File Name: Supplementary Movie 13
Description: 3D rendering of two neurochondrin knockdown indirect flight muscle Z-disks
