# Supplementary material for: Regulation of the evolutionarily conserved muscle myofibrillar matrix by cell type dependent and independent mechanisms
Source: Nat Commun. 2022 May 13;13:2661. doi: 10.1038/s41467-022-30401-9 (PMC9106682; doi:10.1038/s41467-022-30401-9)
Supplement: Supplementary file 17 — Reporting Summary [file 41467_2022_30401_MOESM17_ESM.pdf]

nature portfolio

Corresponding author(s): Brian Glancy
Last updated by author(s): 04/26/2022

Reporting Summary

Nature Portfolio wishes to improve the reproducibility of the work that we publish. This form provides structure for consistency and transparency in reporting. For further information on Nature Portfolio policies, see our Editorial Policies and the Editorial Policy Checklist.

Statistics

For all statistical analyses, confirm that the following items are present in the figure legend, table legend, main text, or Methods section.

n/a	Confirmed	
☐	☒	The exact sample size (*n*) for each experimental group/condition, given as a discrete number and unit of measurement
☐	☒	A statement on whether measurements were taken from distinct samples or whether the same sample was measured repeatedly
☐	☒	The statistical test(s) used AND whether they are one- or two-sided<br>*Only common tests should be described solely by name; describe more complex techniques in the Methods section.*
☐	☒	A description of all covariates tested
☐	☒	A description of any assumptions or corrections, such as tests of normality and adjustment for multiple comparisons
☐	☒	A full description of the statistical parameters including central tendency (e.g. means) or other basic estimates (e.g. regression coefficient) AND variation (e.g. standard deviation) or associated estimates of uncertainty (e.g. confidence intervals)
☐	☒	For null hypothesis testing, the test statistic (e.g. *F*, *t*, *r*) with confidence intervals, effect sizes, degrees of freedom and *P* value noted<br>*Give P values as exact values whenever suitable.*
☒	☐	For Bayesian analysis, information on the choice of priors and Markov chain Monte Carlo settings
☒	☐	For hierarchical and complex designs, identification of the appropriate level for tests and full reporting of outcomes
☒	☐	Estimates of effect sizes (e.g. Cohen's *d*, Pearson's *r*), indicating how they were calculated

Our web collection on statistics for biologists contains articles on many of the points above.

Software and code

Policy information about availability of computer code

Data collection	ZEISS Crossbeam 540 with ZEISS Atlas 5 software, QuantStudio 3 Real-Time PCR System,
Data analysis	ImageJ v1.53p, Ilastik 1.3.3post2, Imaris 9.7.0, Excel 2016, Prism 9.0.0, ZEN 3.2.0.115

For manuscripts utilizing custom algorithms or software that are central to the research but not yet described in published literature, software must be made available to editors and reviewers. We strongly encourage code deposition in a community repository (e.g. GitHub). See the Nature Portfolio guidelines for submitting code & software for further information.

Data

Policy information about availability of data

All manuscripts must include a data availability statement. This statement should provide the following information, where applicable:

- Accession codes, unique identifiers, or web links for publicly available datasets
- A description of any restrictions on data availability
- For clinical datasets or third party data, please ensure that the statement adheres to our policy

Several raw FIB-SEM datasets are freely available at https://doi.org/10.5281/zenodo.5796264. Additional raw data used in this work are available upon reasonable request.

Field-specific reporting

Please select the one below that is the best fit for your research. If you are not sure, read the appropriate sections before making your selection.

☒ Life sciences ☐ Behavioural & social sciences ☐ Ecological, evolutionary & environmental sciences

For a reference copy of the document with all sections, see nature.com/documents/nr-reporting-summary-flat.pdf

Life sciences study design

All studies must disclose on these points even when the disclosure is negative.

Sample size	Sample sizes were chosen based on previous studies using similar measurements in Bleck et al. Nat Comms, 2018 and Willingham et al. Nat Comms, 2020.
Data exclusions	No data were excluded
Replication	All studies were successfully repeated in at least three biological replicates
Randomization	Not applicable due to the lack of perturbations to randomize
Blinding	No blinding was performed due to the clear phenotype differences among muscle types.

Reporting for specific materials, systems and methods

We require information from authors about some types of materials, experimental systems and methods used in many studies. Here, indicate whether each material, system or method listed is relevant to your study. If you are not sure if a list item applies to your research, read the appropriate section before selecting a response.

Materials & experimental systems

n/a	Involved in the study	
☐	☒	Antibodies
☒	☐	Eukaryotic cell lines
☒	☐	Palaeontology and archaeology
☐	☒	Animals and other organisms
☒	☐	Human research participants
☒	☐	Clinical data
☒	☐	Dual use research of concern

Methods

n/a	Involved in the study	
☒	☐	ChIP-seq
☒	☐	Flow cytometry
☒	☐	MRI-based neuroimaging

Antibodies

Antibodies used	Rabbit anti-salm-1 (1:500, gift from Dr. Tiffany Cook, original source: Kuhnlein et al. EMBO, 1994), Rabbit anti-nmr1 (H15) (1:200, gift from Dr. James B. Skeath), and Alexa-Fluor-488-labeled anti-rabbit IgG (1:500, Cat# A32731, Thermo Fisher Scientific).
Validation	The salm and H15 primary antibodies were verified in knockdown experiments from Katti et al. bioRxiv, 2021 (AKA NCOMMS-20-07979A-Z)

Animals and other organisms

Policy information about studies involving animals; ARRIVE guidelines recommended for reporting animal research

Laboratory animals	Drosophila strains and genetics. Genetic crosses were performed on yeast corn medium at 22°C. W1118 were used as controls for the respective genetic backgrounds. Mef2- Gal4 (III chromosome, BS# 27390), 1151-Gal4;UAS-Dicer2 (I, BS# 27390), Act88F-Gal4 (III, BS# 38461), and Mhc-Gal4 (III, BS# 55133) were used to drive muscle specific gene knockdown of respective genes. UAS-mito-GFP (II, BS# 8442) was used as a UAS control. RNAi lines for knockdown of salm (UAS-salm RNAi, V101052), H15 (UAS- H15 RNAi, V28415), and Neurochondrin (NCDN) (UAS-NCDN RNAi, V109002) were purchased from the Vienna Drosophila Resource Center. UAS-NCDN (Neurochondrin) transgenic flies were generated using the plasmid containing BDGP Tagged NCDN ORF (UFO10226; obtained from DGRC). We confirmed the fragment size by performing digestion with EcoRI and further amplified the fragment by PCR and verified by sequencing. The UAS-NCDN transgenic flies were generated using phiC31-mediated integration into the attP2 landing site, and the injection of embryos was performed by Bestgene Inc (USA). All other stocks were obtained from the Bloomington (BS#) Drosophila Stock Center. All chromosomes and gene symbols are as mentioned in Flybase (http://flybase.org). 2-3 day old, male and female flies were used in all experiments except the pupal development data where ages are described (Figure 5f-u).
Wild animals	No wild animals were used

Field-collected samples	No field collected samples were used
Ethics oversight	No ethical approvals are required for Drosophila studies

Note that full information on the approval of the study protocol must also be provided in the manuscript.
